# Preclinical Study of Ibuprofen Loaded Transnasal Mucoadhesive Microemulsion for Neuroprotective Effect in MPTP Mice Model

**Published:** 2018

**Authors:** Surjyanarayan Mandal, Snigdha Das Mandal, Krishna Chuttani, Krutika K Sawant, Bharat Bhushan Subudhi

**Affiliations:** a *School of Pharmaceutical Sciences, Siksha ‘O’ Anusandhan University, Khandagiri Square, Bhubaneswar, Orissa, India. *; b *Department of Pharmacology, Parul Institute of Pharmacy and Research, Vadodara, Gujarat, India.*; c *Division of Cyclotron and Radiopharmaceutical Sciences, Institute of Nuclear Medicine and Allied Sciences (INMAS), DRDO, Delhi-110054, India. *; d *Department of Pharmaceutics, MS University, TIFAC Core, Vadodara, India.*

**Keywords:** Response surface methodology (RSM), Substantia nigra, Flux, Capmul MCM, MPTP, Male C57BL/6 mice, Immunohistochemistry

## Abstract

Ibuprofen, a non-steroidal anti-inflammatory drug (NSAID), showed very promising neuroprotection action, but it suffers from high first pass metabolism and limited ability to cross blood brain barrier. Severe gastric toxicity following oral administration further limits its utility. Hence, the aim of this study was to investigate whether ibuprofen loaded mucoadhesive microemulsion (MMEI) could enhance the brain uptake and could also protect the dopaminergic neurons from MPTP-mediated neural inflammation. In this work, ibuprofen loaded polycarbophil based mucoadhesive microemulsion (MMEI) was developed by using response surface methodology (RSM). Male C57BL/6 mice were intranasally given 2.86 mg ibuprofen/kg/day for 2 consecutive weeks, which were pre-treated with four MPTP injections (20 mg/kg of body weight) at 2 h interval by intraperitoneal route and immunohistochemistry was performed. Globule size of optimal MMEI was 46.73 nm ± 3.11 with PdI value as 0.201 ± 0.19. Histological observation showed that optimal MMEI was biocompatible and suitable for nasal application. The result showed very significant effect (*p < *0.05) of all three independent variables on the responses of the developed MMEI. Noticeable improvement in motor performance with spontaneous behavior was observed. TH neurons count in substantia nigra with the density of striatal dopaminergic nerve terminals after MMEI administration. Results of this study confirmed neuroprotection action of ibuprofen through intranasal MMEI against MPTP induced inflammation in dopaminergic nerves in animal model and hence, MMEI can be useful for prevention and management of Parkinson disease (PD).

## Introduction

There is growing body of literature suggesting the protective role of non-steroidal anti-inflammatory drugs (NSAIDs) against Parkinson’s disease (PD) (1, 2). They can either delay or prevent the onset of PD (3). Since neuroprotective efficacy of individual NSAID vary across PD models in different investigations, no specific NSAID has emerged as unequivocal neuroprotectant against PD. However, in inflammation and stress mediated PD, ibuprofen has exhibited higher capacity for neuroprotection amongst NSAIDs (4-6). Although detail mode of action is yet to be established, it is believed to exert neuroprotection by its anti-inflammatory PPARγ agonistic properties and not by its cox-inhibitory properties (4, 5). This encourages application of ibuprofen for neuroprotection against PD. However, there are several huddles limiting its application like extensive first pass metabolism and limited access through blood brain barrier (7). These factors cumulatively contribute lower bioavailability of ibuprofen in brain. It also suffers from severe gastric toxicity and hence cannot be administered in higher doses or for longer duration as desirable for PD. To overcome this, several attempts have been made either to mask its toxicity or improve its brain availability through prodrug and codrug approach (8, 9). However these efforts have not successfully enabled use of ibuprofen against PD. Thus, there is an unmet need to develop suitable drug-product to extend the application of ibuprofen for neuroprotection against PD.

In recent years, nasal route of drug delivery has become a versatile means of drug administration because of rapid drug absorption, hepatic first-pass metabolism avoidance and more brain uptake of drug, preferentially through the olfactory path (10). Therefore, the nasal route of ibuprofen delivery can overcome the challenges gastric toxicity and limited availability. But, nasal delivery of ibuprofen poses different challenges including nasal mucociliary clearance, metabolism in nasal mucosa and limited nasal volume (11). Maintaining appropriate viscosity is vital to overcome rapid nasal clearance (12). To address these issues we have earlier developed nasal formulations of ibuprofen and evaluated them *in-vitro* (data accepted but not published). Although these formulations showed, indirect evidences of neuroprotection, there were stability issues with these formulations, which prompted us to further our investigation to develop a novel formulation and to generate direct evidence of its action for dopaminergic neuroprotection.

Poor aqueous solubility of ibuprofen is an issue for development of nasal formulation. Making o/w microemulsion (ME) can benefit by improving the aqueous solubility of ibuprofen as only volume of ≤ 400 μL (200 μL/nostril) can be accommodated. Microemulsions are thermodynamically stable, transparent and isotropic liquid mixture of oil, water, surfactant and cosurfactant with a droplet size usually in the range of 10-200 nm. These systems are currently of great scientific interest to the researchers due to their ability to accommodate both hydrophilic and hydrophobic drug molecules (13). Moreover, these versatile delivery systems may enhance the bioavailability of drug by providing protection against oxidation, enzymatic hydrolysis and hence they are useful for drug delivery through almost all routes like oral, ophthalmic, intravenous, pulmonary and topical routes (14, 15). Since last decade, MEs have been used as a vehicle for brain targeting of drugs capitalizing on the nasal route. Inclusion of mucoadhesive agent such as polyelectrolyte polymer is believed to increase the retention time due to the electronic interaction between mucin of nasal mucosa and the polymer of formulation, which facilitates the absorption (16, 17). It is desirable for intranasal delivery to optimize several parameters to have low globule size to ensure higher release and adequate permeation rate. Thus, Box-Behnken experimental design was used in this study to develop an optimal microemulsion formulation by interpreting the influence of formulation compositions on globule size, flux, viscosity, mucoadhesive potential, and % drug release (18, 19).

Hence, the present investigation involves development of transnasal mucoadhesive microemulsion of ibuprofen (MMEI) and evaluation of its compatibility and efficacy to protect against inflammation mediated dopaminergic neurodegeneration. 

## Experimental

*Materials*

Ibuprofen was obtained from Abbott pharmaceutical Ltd. (Goa, India) as gift sample. Labrafil M 1944 CS, Labrafac PG, Labrafil M 2125CS and Transcutol P were received from Gattefosse, France as gift sample. Capmul MCM, Capmul MCM EP and Accenon CC were procured as gift sample from Abitec Corporation, Jackson St Janesville, USA. Colorcon Asia (Mumbai, India) gifted Cremophor RH 40 and Cremophor EL. MPTP-HCl and anti-mouse TH were purchased from Sigma-Aldrich (St. Louis MO, USA). Isopropyl myristate, Tween 60, Tween 80, Isopropyl alcohol Methanol and Triton X-100 were purchased from Gujarat chemical corporations (Vadodara, India).Oleic acid and diaminobenzidine were purchased from Chemdyes (Mumbai, India). All other chemicals were reagent grade. 

*Selection of formulation compositions*

Selection of oil as internal phase for o/w microemulsion is mainly based on the solubility of drug. Different oils like Isopropyl Myristate, Oleic acid, Capmul MCM EP, Capmul MCM, Labrafil M 1944CS, Labrafac PG and Labrafil M 2125CS were screened for solubility study. For solubility study as described in the literature, excess of ibuprofen was added to each capped vial containing 5 mL of the selected oils separately. After sealing, mixtures were shaken at suitable rate with orbital shaker at 37 ± 2 °C for 48 h. After reaching equilibrium, each vial was centrifuged at 5000 rpm for 10 min and excess insoluble ibuprofen from the supernatant was separated by filtering through 0.45 μm Whatman filter. Drug solubility was quantified from supernatant by UV-VIS Spectrophotometer (Shimadzu UV1800) (20). Selection criteria for surfactant for o/w microemulsion development were HLB value, drug solubility and non-toxic nature. Several non-toxic surfactants like Accenon CC, Cremophor RH 40, Cremophor EL, Tween 60 and Tween 80, having HLB value ranging in between 14 to 18, were screened. Screening of co-surfactants was done on their ability to form stable microemulsion at minimum concentration and several co-surfactants like PEG 400, PEG 600, Propylene glycol, Glycerol, Isobutyl alcohol, Isopropyl alcohol and Transcutol P were screened. 

*Pseudoternary phase diagrams*

Pseudoternary phase diagrams were developed by water titration method using screened oil, mixture of surfactant, co-surfactant (Smix) and water to find the suitable composition ratio. The liquid mixtures of oil and Smix (1:0.5, 1:1, 1.5:1, 2:1, 2.5:1 and 3:1) at certain volume ratio were titrated with different concentrations of aqueous polycarbophil solution. Using Chemix software, microemulsion regions were determined and Smix showing maximum microemulsion region was used for the development of microemulsion (21). 

*Preparation of Formulations and Experimental design*

The concentration of oil, Smix and aqueous polycarbophil solution were selected from pseudoternary phase diagrams and drug solubility data. These values were then manipulated using Design-Expert^® ^software (Stat-Ease, Inc., Minneapolis, Minnesota, USA, Version 7.1.0). Three independent variables *i.e.*, amount of Labrafil M 1944CS (X1), Smix (Tween 80: Transcutol P, X2) and amount of polycarbophil (X3, in terms of % w/v in water) with their three levels was taken from the data of preliminary experiments. Total 15 MMEIs was obtained. A suitable polynomial model was selected experimentally basing on significant terms (*p *< 0.05), non-significant lack of fit, multiple correlation coefficient (r^2^) and adjusted multiple correlation coefficient (adjusted r^2^) data as provided by Design-Expert^®^ software (22). The design of the experimental was quadratic and details of the levels as taken are demonstrated in Table 2. MMEIs were prepared experimentally using the compositions of all model MMEIs as summarized in Table 2. Smix *i.e.*, mixture of Tween 80 and Transcutol P (3:1) was mixed well with drug dissolved Labrafil M 1944CS solution. The above mixture was then titrated with different aqueous polycarbophil concentration with mild and continuous stirring by magnetic stirrer at room temperature (20, 21). Finally average globule size, flux, viscosity, mucoadhesive potential (residence time) and % drug release were determined experimentally for all 15 formulations. Flux was quantified from the *ex-vivo* permeation study. Plain ibuprofen gel (IPG, 3.0 mg/mL) was prepared by dispersing ibuprofen (30 mg) to the already prepared 0.5% aqueous based plain polycarbophil based gel with continuous stirring. pH of the final formulations was also checked. 

*Optimization*

Responses like average globule size, flux, viscosity, mucoadhesive potential, and % drug release were selected for both numerical and graphical optimization. It was decided to choose maximum of flux and % drug release while minimum of average globule size with suitable viscosity and mucoadhesive potential in order to obtain a final optimized formulation. On a contour plot, visually search for the best compromise which stands for the formulation with desirable values for all responses was done. Finally for verification, check point batches were prepared experimentally and all five responses were compared *i.e.*, predicted v/s observed value as shown in Table 4. Best suited composition was considered as optimized batch and was used for further study.

*Evaluation of dependent variables*

Globule size of optimized MMEI was determined by Zetasizer (Nano ZS; Malvern Instruments Inc, Malvern, UK). The viscosity was determined using Brookfield viscometer (HVDVII, USA). The sample was sheared using spindle No. 62 at 30 rpm and at room temperature and the result were taken after the stabilization of the display. All experiments were repeated for three times (21). Zeta potential of optimal MMEI was also determined by Zetasizer using the electrophoretic mobility.

The mucoadhesive potential which indicated by the residence time of developed nasal formulations was evaluated as per reported method (21). Briefly, 100 mg MMEI was kept on the center of the separate agar plates at room temperature (1% w/w, prepared in PBS, pH 6.4). After 10 min, the agar plates were attached to USP disintegration test apparatus and allowed to move up and down at rate 30 ± 2 in PBS at 37 ± 2 °C. The time taken by the formulations to separate from the agar plates was noted visually as residence time of the formulations.

*In-vitro* drug release of developed MMEI was performed using modified dissolution apparatus. Five mL of MMEI (≈ 15 mg of ibuprofen) was taken in dialysis membrane (10,000 D) and was kept in 200 mL of the dissolution medium *i.e.*, phosphate buffer, pH 6.4 equilibrated at 37 ± 0.5 °C and 50 rpm (23). Comparative release profile was studied for developed MMEI and IPG. At predetermined time interval, 5mLaliquots were withdrawn from receptor compartment and then replaced with same volume of freshly prepared dissolution medium equilibrated at 37 ± 0.5 °C. The samples were analysed for drug release by UV-VIS Spectrophotometer (23). 

The drug permeability through excised sheep nasal mucosa was determined using Franz diffusion cell with an effective diffusion area of 7.06 cm^2^ and volume 30 mL (24). The prepared nasal mucosa with same thickness was mounted on to the receptor compartment having already 30 mL of diffusion medium, phosphate buffer (pH 6.4). The donor compartment was assembled to it and was loaded with 1 mL of MMEI and IPG (≈ 3 mg of ibuprofen). Diffusion was done at 37 ± 0.5 °C and 50 rpm. At predetermined intervals of 10 min, an aliquot of 0.5 mL was withdrawn from the receptor medium and was analyzed by UV-VIS Spectrophotometer. Each obtained data point represented the average of three determinations. Flux (amount drug release per unit area of the sheep nasal mucosa) was determined (25).

*Data Analysis*

Effect of all independent variables on the taken responses like average globule size, viscosity, flux, mucoadhesive potential and % drug release for all MMEIs were treated by using Design-Expert^® ^software. The linear, quadratic and cubic models used to deduce the relation between independent and dependent variables were shown in Table 3. The terms with *p*-value of less than 0.05 were considered as significant terms. The suitability of the models was decided by comparing several statistical parameters like *p*-value of the model (*p*-value must be less than 0.05), *p*-value of lack of fit (greater than 0.05), the multiple correlation coefficient (*R*^2^), adjusted multiple correlation coefficient (adjusted *R*^2^) and the coefficient of variation obtained from the experimentation using Design-Expert^®^ software (26, 27).

*Nasal Ciliotoxicity*

Nasal ciliotoxicity study was done to describe the suitability of the developed formulation. In brief, first male C57BL/6 mice weighing 25-40 g were anesthetized using intra-peritoneal injection of ketamin (45 mg/kg) and acepromazine (1 mg/kg) and then developed MMEI at a dose of 2.86 mg/kg ibuprofen was administered through the intranasal route. Then the animals were sacrificed after 2 h of intranasal drug treatment and the nasal mucosal part from the bottom of inferior meatus was dissected out carefully. The tissues were immediately immersed in 10% neutral formalin and finally mucocilia examination was performed with an optical microscope (Nikon Fx-35A, Japan). Saline and propranolol (a serious nasal mucociliary toxicity agent, 1% w/v solution) were used as a negative and positive control, respectively for this study (28). 

**Table 1:** Ibuprofen solubility in different screened vehicles of microemulsion formulation.

Vehicle Type	Name of the component	Solubility (mg/mL)^a^
Oil	Isopropyl Myristate	12.44 ± 2.63
	Capmul MCM EP	19.54 ± 2.54
	Capmul MCM	29.31 ± 3.13
	Labrafac PG	26.47 ± 1.64
	Oleic acid,	11.24 ± 1.97
	Labrafil M 1944CS	26.59 ± 2.66
	Labrafil M 2125CS	29.3 ± 3.13
Surfactant	Accenon CC	17.29 ± 2.51
	Cremophor RH 40	18.88 ± 1.98
	Cremophor EL	15.76 ± 2.11
	Tween 60	14.61 ± 2.34
	Tween 80	11.29 ± 2.66

a Data expressed, mean ± SD, n = 3.

**Table 2:** Details of Variables with levels, compositions and responses of MMEI model formulations provided by Box-Behnken Design

Batch	X1	X2	X3	Globule Size (nm)	Viscosity(Ps)	Flux (µg/cm^2^. h)	RT(min)	% Drug Release
F1	0.10	1.53	0.50	55.55	25.5	15.8	17.2	96.1
F2	0.50	1.53	0.50	64.29	30.6	11.2	21.5	83.1
F3	0.10	1.65	0.50	58.88	34.6	16.3	23.4	80.0
F4	0.50	1.65	0.50	54.46	31.9	12.4	22.1	86.2
F5	0.10	1.60	0.25	54.23	22.8	15.4	16.2	90.4
F6	0.50	1.60	0.25	65.78	31.7	17.3	21.8	76.4
F7	0.10	1.60	0.75	79.89	36.2	14.6	24.7	86.2
F8	0.50	1.60	0.75	88.79	39.3	14.1	27.8	79.3
F9	0.30	1.53	0.25	58.64	29.5	16.4	21.1	85.8
F10	0.30	1.65	0.25	52.61	35.5	24.2	23.7	90.4
F11	0.30	1.53	0.75	72.44	33.9	18.3	23.2	87.3
F12	0.30	1.65	0.75	61.16	34.1	22.0	23.1	85.7
F13	0.30	1.60	0.50	52.47	31.9	26.1	22.6	88.3
F14	0.30	1.60	0.50	53.11	31.8	26.3	22.5	88.6
F15	0.30	1.60	0.50	52.49	32.6	26.4	22.4	88.1
**Variables**						**Low **	**Medium **	**High**
X1 = Labrafil M 1944 CS (Oil)						0.1 mL	0.3 mL	0.5 mL
X2 = Tween 80: Transcutol P (Smix)						1.55	1.60	1.65
X3 = % Polycarbophil						0.25%	0.5%	0.75%

Amount of ibuprofen = 30 mg; Total volume of MMEI = 10 mL.

RT: Retention time (time required for formulations to separate from the agar plates).

**Table 3:** Statistical parameter of responses determined by Multiple Regression Analysis

Regression Coefficient	Coefficient Estimate				
	Globule Size (nm)	Viscosity (Ps)	Flux (µg/cm^2^. h)	RT (min)	% Drug Release
A- Labrafil M 1944 CS (X1)	1.89	0.31	-0.88	1.19	-2.58
B- Smix (X2)	-3.98	0.26	1.52	-0.21	1.87
C- % Polycarbophil (X3)	5.71	1.87	-1.17	3.55	-2.63
AB (X1 X2)	–0.58	0.68	0.27	0.25	0.88
AC (X1 X2)	0.89	2.15	- 1.05	2.79	–1.95
BC (X1 X2)	2.54	–0.78	–0.43	0.27	–1.89
A^2^	1.66	0.44	–0.45	0.87	1.34
B^2^	–1.54	–0.28	0.83	-0.76	- 0.67
C^2^	3.88	1.41	-1.55	1.04	-1.34
Model (*p*-Value)	0.0003	0.0002	0.0001	0.0021	0.0001
Coefficient of variation	3.18%	2.27%	2.11%	8.40%	2.37%
R^2^	0.991	0.977	0.999	0.962	0.999
Adjusted R^2^	0.973	0.947	0.998	0.976	0.997
Lack of Fit (*p*-Value)	0.540	0.088	0.289	0.877	0.0580

**Table 4:** Results of size, viscosity, flux, retention time (RT) and % drug release of formulation based on their predicted and observed data

No	Components			Globule Size (nm)		Viscosity (Ps)		Flux [µg/cm2h]		RT (min)		% Drug release	
	O	Smix	P	Pre.	Obs.	Pre.	Obs.	Pre.	Obs.	Pre.	Obs.	Pre.	Obs.
1	0.30	3.80	0.50	48.6	47.4	39.8	38.2	24.0	23.9	22.9	22.2	93.9	95.6
2	0.31	3.80	0.52	48.4	47.5	39.5	37.1	24.7	23.8	22.8	22.0	89.8	93.0
3	0.31	3.75	0.50	48.9	48.0	39.5	38.0	25.1	24.5	22.9	22.1	89.4	92.6
4	0.30	3.70	0.50	46.7	46.3	39.3	38.7	25.4	25.8	22.9	22.8	94.1	94.8
5	0.34	3.80	0.50	46.6	48.1	40.2	39.5	25.7	24.9	22.8	22.5	88.6	92.4

O: Oil; Smix: Mixture of surfactant and co-surfactant; P: Polycarbophil

(Pre.: Predicted and Obs.: Observed)

**Table 5:** Effect of ibuprofen on dopamine and its metabolites with turnover rate after 21 days of study.

Groups	DA	DOPAC	HVA	Turnover (DOPAC + HVA/DA)
	Measurement in (μg/g tissue)			
Group 1 (Normal control)	12.92 ± 0.88	2.93 ± 0.38	1.83 ± 0.43	0.36 ± 0.08
Group II (MPTP control)	5.47 ± 0.73^#^	0.98 ± 0.19^#^	0.28 ± 0.08^#^	0.23 ± 0.05^#^
Group III (MPTP-MMEI)	9.76 ± 0.58**	1.85 ± 0.23**	1.44 ± 0.33**	0.33 ± 0.08**
Group IV (MPTP-IDS)	5.49 ± 0.44*	1.09 ± 0.17*	0.47 ± 0.17*	0.28 ± 0.08*
Group V (ORAL-IDS)	6.44 ± 0.26	1.01 ± 0.14	0.51 ± 0.22*	0.23 ± 0.07*

Symptomatic effect of ibuprofen against MPTP-induced dopaminergic neural toxicity. Intranasal administration of ibuprofen significantly protected the depletion of dopamine with concomitant elevation in DA turnover in MPTP-induced mice. Values are expressed as mean ± SD and statistically significant (#*p *< 0.05) for normal control v/s MPTP and

(** *p *< 0.05) MPTP v/s ibuprofen treated groups (n = 06).

**Figure 1:**
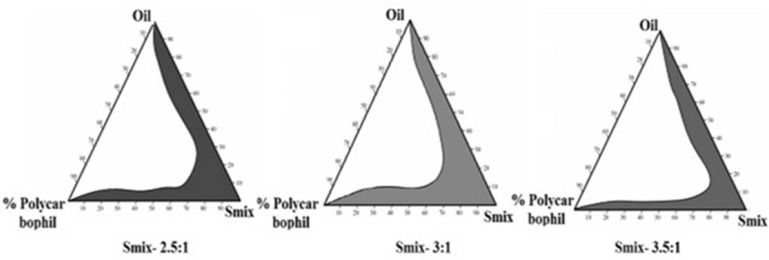
Pseudo-ternary Phase diagram of MMEI showing microemulsion existing region with 3:1 Smix.

**Figure 2:**
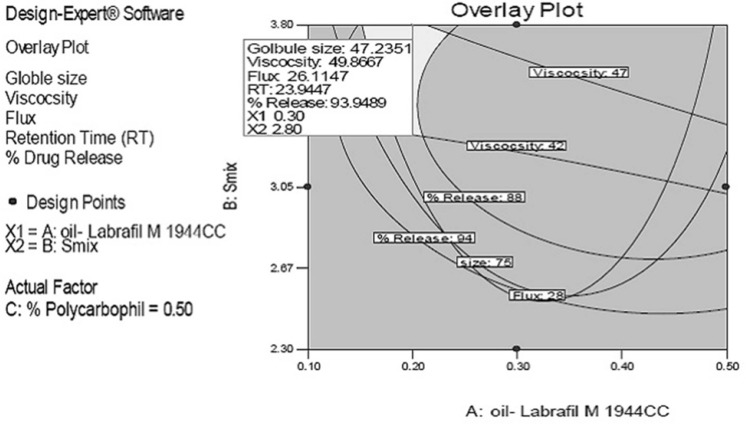
Overlay plots of responses like globule size, viscosity, flux, retention time and % drug release of predicted formulations

**Figure 3:**
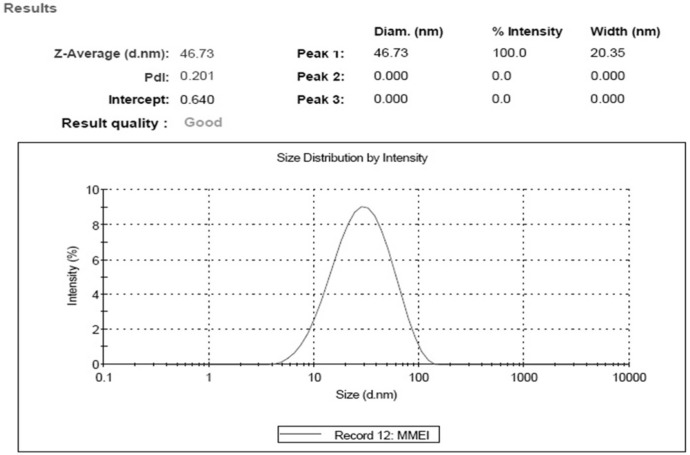
Result of globule size and size distribution indicating the nano size range of developed MMEI.

**Figure 4.**
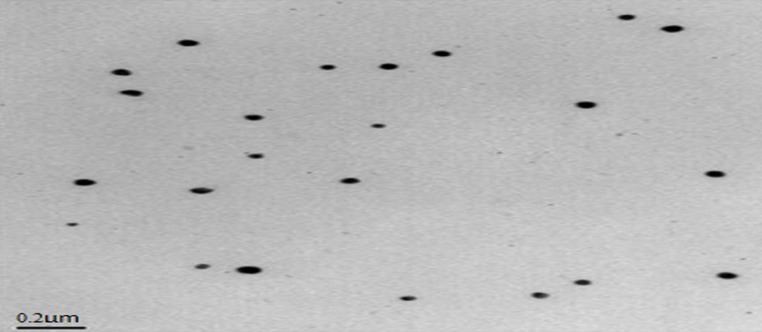
TEM result of the optimized MMEI indicating the narrow particle size with uniform distribution

**Figure 5:**
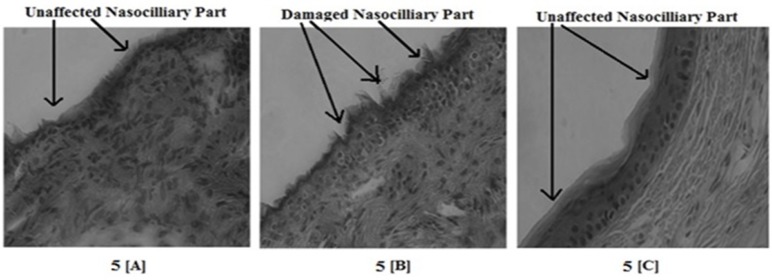
Result of nasociliotoxicity study showing the nontoxicity of developed MMEI. 5[A], 5[B] and 5[C] are representative of Saline, Propranolol and developed MMEI treated mucosal part respectively

**Figure 6:**
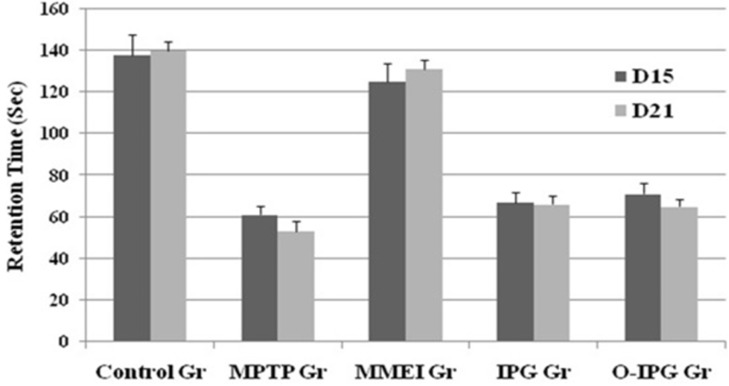
Effect of ibuprofen through MMEI on motor coordination in a rota-rod test.

Data are expressed as mean ± SD (06 animals per group).

**Figure 7.**
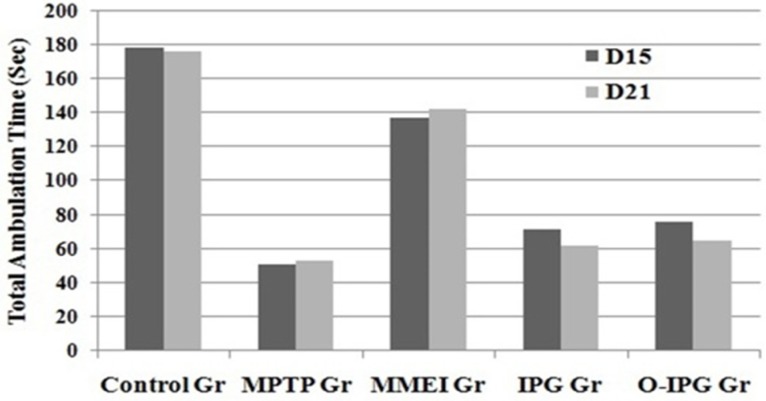
Effect of ibuprofen through MMEI on gross behavior as assessed through an open field test. Values are expressed as mean ± SD (n = 06 animals per group).

**Figure 8.**
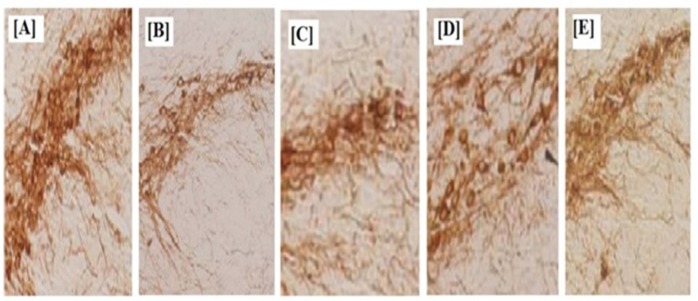
Effects of ibuprofen on MPTP-induced DA terminal loss in striatum and dopaminergic neuronal death in Substantia nigra, [A] Saline [B] MPTP treated Mice, [C] and [D] ibuprofen treatment after 15^th^ day and 21^st^ day, [E] Treated with IPG. Normal control *vs* MPTP (#*p *< 0.05) and MPTP *vs* treated group (*p < 0.05

*In-vivo experimental study*

This animal study was approved by the Institutional Animal Ethical Committee (CPCSEA No. 927/AC/06/CPCSEA), Government of India, New Delhi, India. In the present study, male C57BL/6 mice (20–25 g; 8–12 weeks old) were procured and were kept in animal house. Temperature (25 ± 2 °C) and humidity (60 ± 5 %) condition was maintained throughout the study. Test animals were divided to four groups (six animals per group) and were supplied with standard laboratory diet and reverse osmosis (RO) water ad libitum on a 12 h light/dark cycle. Group I (Normal Control) mice were treated nasally with saline (0.9 % w/v of NaCl). Group II (MPTP control) mice were treated with MPTP at a dose of 4 × 20 mg/kg/day. Two mg/mL of MPTP-hydrochloride saline solution was prepared using 0.9 % w/v aqueous NaCl solution and four doses of 20 mg/kg of animal was intraperitoneally administered at two h intervals (total dose of 80 mg/kg of body weight). For Group III (MMEI treated group) mice were first treated with MPTP (4 × 20 mg/kg/day) in the same manner as that of Group II followed by intranasal administration of optimized MMEI at 2.86 mg/kg of ibuprofen for 7 consecutive days. Similarly, Group IV (Gel treated group) mice were first treated with MPTP (4 × 20 mg/kg/day) followed by intranasal administration of IPG (same concentration that of MMEI) at 2.86 mg/kg of ibuprofen for 7 consecutive days.

Thirty-five to forty μL of developed nasal MMEI containing ibuprofen equivalent to 2.86 mg/kg were administered to the turbinates of nasal mucosa with the help of a micropipette (200 μL) attached to low density polyethylene tube with 0.1 mm as internal diameter at the delivery site (29). Ventral mid brain and striatum of the experimental animals were dissected at 7^th^ day and 15^th^ day after the ibuprofen dose (15^th^ day and 21^st^ day of study) by euthanized with ketamine and then the brain tissues were preserved at -80 ± 1 °C for further investigation.

*Determination of muscular coordination by Rota-rod test*

The muscular or motor coordination was assessed as described in the literature (30). Rota-rod apparatus (Baroda Int. Pvt. Ltd. India) with four rotating rod of 30 mm diameter was used in this study. The apparatus was designed to record the time automatically when a mouse falls off from the rotating shaft. Six mice from each four groups at a time were used. The time spent in seconds by each mouse on the rotating rod at 16 rpm (total duration of study was 180 sec) recorded as a measure of motor function at 15^th^ and 21^st^ day of the study. 

*Assessment of spontaneous activity by open field test*

Comparative spontaneous behaviour of the mice was assessed by open field test as described by Michele and coauthors (31). The open-field apparatus was a fabricated box made up of clear plexi-glass with dimension of 26 × 26 × 39 cm. The floor was lined by equal segments and sensor system consisting of 16 photo beams was installed. Briefly, six animals from each group were placed in the centre of the floor of the box and the spontaneous (the number of crossed segments) behaviour was monitored for 5 min. Fine movement of the mice through the photo beams as expressed as total ambulation time was determined at 15^th^ and 21^st^ day of the study. 

*Assessment of dopamine and its metabolites in mice brain*

Concentration of Dopamine (DA) as well as dihydroxyphenyl acetic acid (DOPAC) and homovanillic acid (HVA) in brain was quantified by HPLC-fluorescence detection as described in the literature (32, 33). Striatum after carefully excised from mice brain washed thrice with saline and was then homogenized in 0.1 M perchloric acid using tissue homogeniser. The homogenate was centrifuged in a refrigerated centrifuge at 15,000 rpm for 20 min at 4 °C and the supernatant was filtered through 0.45 μm membrane filter. Mobile phase used for this HPLC method was methanolic buffered solution of pH 4.5 (0.02 mol/L sodium citrate, 0.05 mol/L sodium dihydrate phosphate) and methanol at 19:1. Twenty µL of the above filtered test sample was injected and the quantification was done at flow rate of 1 mL/min and temperature 25°C. Signals of DA, HVA and DOPAC were detected at 280 nm and 320 nm as the excitation wavelength and an emission wavelength respectively. The quantification of DA, DOPAC and HVA was done from the obtained chromatographic peak areas and results were expressed as µg/g of striatal tissue at 15^th^ and 21^st^ day of the study.

*Immunohistochemistry Study*

Immunohistochemistry study as a direct evidence of the neuroprotection was performed as per the procedure described in literature (34, 35). In brief, substantia nigra (Sn) and striatum region of mice brain were excised carefully by opening the skull. Slides were prepared in paraffin using xylene, rehydrated with absolute alcohol and were then boiled for 20 min in citrate buffer (10 mM, pH 6) to nullify the interference of endogenous peroxidase. The slides were kept in buffer *i.e.*, 10% normal sheep serum (NGS) with 0.2% Triton X-100 in 0.01M PBS (TTBS) at 37 C for 30 min to completely block the activity of peroxidase and then washed three times with 0.01M PBS for 10 min. Further incubation of sections was done with anti-mouse TH (1: 1000) in 2% NGS and 0.2% TTBS for 24 h. After washing with 1% TBS, the sections were further subjected to secondary incubation with anti-mouse IgG-HRP conjugated secondary antibody (1: 1000 in 1.5% NGS) for 2 h. The incubated sections were washed with PBS to remove the antibody and final incubation was done with diaminobenzidine (DAB) to view before analyzing the TH immunoreactivity. The intensity of TH immunoreactivity in striatum was measured and results are expressed as percentage of control. Numbers of TH immunoreactive neurons in Sn were then counted at higher magnification (45x). TH neuron count in Sn and the density of striatal dopaminergic nerve terminals were determined by light microscope (Olympus, Tokyo, Japan). 

*Statistical analysis*

All obtained data are reported as mean ± SD. Difference between the groups were quantified using Student’s *t*-test at the significant level *i.e.*, *p *< 0.05. More than two groups were compared in each test point using ANOVA and significant data was set at *p *< 0.05. 

## Results and Discussion

*Selection of formulation compositions*

As shown in Table 1, Labrafil M 1944CS was showed comparatively higher ibuprofen solubility (29.3 ± 3.13 mg/mL) than other screened oils and was selected as oil phase for this study. But, Tween 80 with HLB value 15.0 and relatively less drug solubility (11.29 ± 2.66 mg/mL) than other screened surfactants, was selected as surfactant (Table 1).

Since drug solubility was more in selected oil than that of surfactant, more control over the drug release was observed due to the reservoir nature of the oil phase and less amount of oil was required to accommodate drug for nasal delivery. So, relatively less surfactant and cosurfactant will be required to stabilize the oil to develop a more suitable microemulsion system. Pseudo-ternary phase diagrams as shown in Figure 1 showed relatively more microemulsion zone was observed with Smix (3:1). More microemulsion zone indicated more stability with less bi-continuous phase and was thus used to develop microemulsion formulation (21).

*Formulation development*

In order to assess the effect of independent variables on the responses like average globule size, viscosity, flux, mucoadhesive potential and % drug release, three independent variables (X1, X2, and X3) were statistically analyzed based on the response surface method (RSM) using Design-Expert^® ^software and the results of multiple regression analysis are summarized in Table 2.

It was also observedas shown in Table 3, average globule size, viscosity, flux, mucoadhesive potential and % drug release of ibuprofen was noticeably influenced by the independent variables and their interaction effect which were illustrated in the polynomial Equations 1 to 5.

Analysis for all responses indicated the suitability of quadratic model *(p *< 0.05*)*. By running ANOVA, the final equations for all responses *i.e.*, average globule size, viscosity, flux, mucoadhesive potential and % drug release in their respective coded value were obtained as follows. 

Globule Size = + 66.87 + 1.89 × A – 3.98 × B + 5.71 × C – 0.58 × (A × B) + 0.89 × (B × C) + 2.54 × (A × C) + 1.66 × A^2^ – 1.54 × B^2^ + 3.88 × C^2^                     (1)

Equation 1 showed average globule size of MMEI was mainly influenced by Labrafil M 1944CS, Tween 80 - Transcutol P concentration ratio and polycarbophil with their interactions. For development of an effective intranasal drug delivery system, globule size plays vital role as it influences the *in-vivo *absorption of drug from the formulations (36). The globule size is a crucial characteristic of microemulsion formulation because it influences drug release rate and hence the *in-vivo* profile of the drug. Consequently, optimizing microemulsion with smaller globule size to ensure rapid penetration through the nasomucosal layers was one of the aims of this study. The observed globule size of the formulations ranged from 42 nm to 88 nm (Table 2). 

The influence of the screened variables on the average globule size of MMEI is presented in Equation 1. 

The obtained results in this design indicated that oil concentration (X1) and mucoadhesive polymer concentration (X3) has significant effect on the mean globule size *i.e.*, *p *= 0.0003. As depicted, increasing oil concentration from 0.1 mL to 0.5 mL and mucoadhesive polymer concentration from 0.25% to 0.75% caused a significant increase in average globule size. 

This may be due to the fact that Smix at its increased concentration could able to reduce the interfacial tension between oil and aqueous phase. But at higher amount of oil with same Smix concentration, the hydrophobicity oil was not being masked by Smix, resulting into more interfacial tension and increased globule size. Mucoadhesive polymer also found to increase the globule size which may be due to the fact that it was capable of absorbing water and swell which in turn disturb the hydrophilic-lipophilic balance of the system. 

Viscosity = + 34.44 + 0.31 × A + 0.26 × B + 1.87 × C + 0.68 × (A × B) + 2.15 × (A × C) – 0.78 × (B × C) + 0.44 ×A^2^ – 0.28 × B^2^ +1.41 × C^2^                     (2)

It (Equation 2) suggested that viscosity was mainly influenced by % polycarbophil. Moreover interaction effect of Labrafil M 1944CS and polycarbophil got positive effect on viscosity of MMEI. Viscosity was mainly influenced by % polycarbophil which may be due to the fact that mucoadhesive polymer (polycarbophil) possesses hydrogen bonding group along with its extensive hydration property. Polycarbophil is capable of imbibing relatively more water and increases the viscosity of formulation hence drug release was controlled as such. This may be due to the influx of more dissolution fluid into the formulation.

Flux = + 31.13 - 0.88 × A +1.52 × B - 1.17 × C + 0.27 × (A×B) - 1.05 × (A × C) – 0.43 × (B × C) – 0.45 × A^2 ^+ 0.83 × B^2^ - 1.55 × C^2^                     (3)

Labrafil M 1944 CS due to its reservoir action and Polycarbophil due to viscosity enhancing property showed negative effect on release rate and hence the flux. The results obtained in this design indicated that oil concentration (X1) and mucoadhesive polymer concentration (X3) has significant effect on the flux through sheep nasal mucosa (*p = *0.0001). However, Smix showed positive effect on flux due to the fact that both Tween 80 and Transcutol P capable of altering the permeation behavior of the membrane by changing the fluidization of lipid enabling the drug molecule to permeate through rapidly. 

Retention Time = + 4.18 + 1.19 × A - 0.21 × B + 3.55 × C + 0.35 × (A × B) + 2.79 × (A × C) + 0.29 × (B × C) + 0.87 × A^2 ^– 0.76 × B^2^ + 1.04 × C^2^                     (4)

Labrafil M 1944CS and Polycarbophil showed more positive effect while Smix though non-significant, showed negative effect as shown in Equation 4. Mucoadhesive nature of the polymer may be because of the presence of high density of hydrogen bonding groups which could combine with mucin more strongly as shown in Equation 4 (*p* = 0.0021) (24). Moreover, interaction effect of Labrafil M 1944CS and polycarbophil got positive effect; this may be due to the mucoadhesive and viscosity enhancing property of the polymer. 

% Drug Release = + 74.40 – 2.58 × A + 1.87 × B - 2.63 × C + 0.88 × (A × B) – 1.95 × (A × C) – 1.89 × (B × C) + 1.34 × A^2 ^- 0.67 × B^2 ^- 1.34 × C^2^                     (5)

Drug release was heavily negatively influenced by Labrafil M 1944CS and polycarbophil, while mixture of Tween 80 and Transcutol P showed positive effect as shown in the Equation 5. Drug release after 8 h ranged from 79% to 96% as shown in Table 2. Formulation variables of MMEI influenced the drug release as shown in Equation 5. Drug release was heavily but inversely influenced by Labrafil M 1944 CS and polycarbophil, while mixture of Tween 80 and Transcutol P (3:1) showed positive effect as shown in the Equation 5. Labrafil M 1944 CS decreased the drug release from the formulation due to its reservoir property and polycarbophil because of its viscosity enhancing property. Smix was found to increase ibuprofen release because of the enhanced water solubility of poorly water soluble ibuprofen. This action may be coupled with the water absorption capacity of the screened mucoadhesive polymer *i.e.*, polycarbophil. 

The rationale of optimization through factorial design was to obtain the defined targets for all responses simultaneously with respect to the predefined ones. In this study, globule size, viscosity and retention time (RT indicating the mucoadhesive property) was set to maximum without affecting release while flux and release were set to maximum. Overlay plots of all responses for predicted formulations at three different water contents as the actual factor are depicted in Figure 2. The grey region stands for formulations with minimum globule size, maximum release and maximum flux.

In order to confirm the desirability of the optimized MMEI, five formulations were prepared experimentally and all responses were evaluated as given in Table 3. It was observed that experimentally found data were matching with the predicted responses for all five MMEIs and hence, the optimization process was verified. 

*MMEI characterization*

MMEIs with 0.5% w/v of polycarbophil, 0.3 mL Labrafil M 1944 CS, 3.70 mL of Smix (Tween 80 and Transcutol P at 3:1 ratio) showed the lowest globule size, highest flux, optimum viscosity and highest drug release as shown in Table 4 and hence was considered as optimized formulation.

Optimal MMEI was found to be transparent with globule size 46.73 nm ± 3.11 with PdI value (0.201 ± 0.19) as shown in Figure 3.

PdI value and TEM results as shown in Figure 4 indicated the mono-dispersity of the developed MMEI with nano globule size range. Intranasal permeation of drug is believed to be influenced by monodispered nano formulations (36).

Zeta potential of the formulation was -24.4 mV ± 3.27. Zeta potential indicated stability of formulation as globules did not show intense aggregation or repulsion (37) and hence monodispersity and the shelf life of the formulation will be maintained. Viscosity of MMEI was found to be ranging from 42.37 Ps to 48.37 Ps at 25 °C. Mucoadhesive potential of MMEI in terms of retention time of formulation was found to be 23.1 min ± 1.2 which was adequate enough to get adhere on the nasal mucosa. 

Moreover, the obtained result indicated that physical properties of MMEIs were profoundly influenced by independent variables and their combinations. Developed MMEI through its viscosity data showed to have adequate adhesion property which enhanced the adhesion property of formulation on the nasal mucosa. This again can be considered as a contributing factor for enhancement in the efficacy of MMEI by minimizing the nasal clearance. 

The percentage of drug release showed that 94.77% ± 4.25 of drug was sustained released for 8 h from MMEI. From the data, it was observed that the release rate was decreased with increase amount of independent variables like oil and mucoadhesive polymer while surfactant-cosurfactant mixture was increased the drug release. 

The flux was found to be of 25.82 [µg/(cm^2^ h)] The effect of oil, mixture of surfactant and co-surfactant and polycarbophil on different properties of microemulsion and drug penetration capacity was evaluated by RSM with Box-Behnken design and the results indicated that MMEI system had a significant permeation enhancement effect for intranasal delivery of ibuprofen. This can be attributed to factors including, higher solubility of ibuprofen in oil phase, less solubility in surfactant and optimized viscosity due to polycarbophil (38). The flux data revealed rapid permeation through nasal mucosa which may be due to the relatively smaller globule size and cumulative penetration action of surfactant, co-surfactant and oil by altering the fluidization lipid of nasal mucosa. Moreover, the rapid permeation of ibuprofen through developed MMEI was supported by the enhanced mucoadhesion of the formulation on the nasal mucosa.

*Nasal Ciliotoxicity study*

No mucociliary alteration of mice nasal mucosa was observed following intranasal administration of MMEI (Figure 5) whereas complete destruction of the nasal mucosa was observed with positive control animals. Results of nasal ciliotoxicity did not reveal any toxicity issues which supports the acceptability of MMEI for intranasal application. 

*Assessment of spontaneous activity and muscular coordination*

A noticeable DA turnover elevation was observed for intranasal MMEI treated group compared to MPTP control group (*p *< 0.05). As shown in Figure 6, retention time was found to be 53 to 67 sec for the MPTP treated group compared to 125 to 131 sec for the ibuprofen treated group. Further, a significant reduction in total ambulation time was observed in MPTP-intoxicated group as compared to normal controls. Total ambulation time for the MPTP treated group was around 50 sec, in comparison to 137 to 142 sec for ibuprofen treated group. Moreover the total ambulation time for the MMEI treated group was significantly higher as compared to the MPTP-intoxicated group (122 sec v/s 47 sec).

Ibuprofen treatment (2.86 mg/kg) through intranasal MMEI as in third group of animals significantly (*p *< 0.05) improved the muscular coordination activity as compared to MPTP control as shown in Figure 6. Further, MPTP-intoxicated group showed steep reduction in total ambulation time as compared to ibuprofen treated group as shown in Figure 7.

Since MPTP is a toxin, the reduced level of DA, DOPAC and HVA in MPTP treated animals reflect the inflammation of the dopaminergic nerve endings. So the developed formulation through nasal route bypassed the barriers and reached the neural part with desired concentration to exert the anti-inflammatory action. Elevations of these parameters in MMEI pre-treated (intranasal) animals provide direct evidence of neuroprotection by the MMEI.

*Dopamine and its metabolites assessment in brain*

Striatal DA content after MPTP intoxication decreased to 29.92% (4.68 ± 0.77) which was elevated to 58.21% (9.12 ± 0.58) following administration of MMEIin comparison to normal control (15.67 ± 1.54). So MPTP intoxication significantly (*p *< 0.05) decreased striatal DA contentto less than one third to that of control. In-addition, a concomitant elevation of DA turnover was also observed in MPTP induced mice after 15^th^ day of ibuprofen dose as shown in Table 5.

*Immune-histochemistry study*

Further, in order to generate direct evidence of neuroprotective action, immune-histochemistry study was performed. Density of striatal dopaminergic nerve terminals (TH density in striatum) was found to be 4.5 ± 1.21 for MPTP treated group. TH density in striatum for MMEI treated group was found to be 14.9 ± 1.43 and 22.46 ± 1.27 on 15^th^ and 21^st^ day of the test respectively. MPTP resulted in significant decrease in neural density substantia nigra, when compared to control group (*p *< 0.05). However, nasal administration of ibuprofen through MMEI increased TH expression in substantia nigra and density of striatal dopaminergic nerve terminals as shown in Figure 8 compared to MPTP group (*p *< 0.05). Figure 8B showed complete nerve destruction while regeneration was observed with ibuprofen treated groups. Significant differences was noticed for MMEI treated group as shown in Figures 8C and 8D at 15^th^ and 21^st^ day of study than that of Figure 8E, which is ibuprofen dispersed gel group (IPG). MPTP resulted in a significant decrease in TH density in striatum and TH-positive neurons in Sn as compared with the control group (*p *< 0.05).

This may be due to the fact that MMEI provides control release of ibuprofen while IPG. The mechanisms of the neuroprotective effects ibuprofen have earlier been linked to its anti-inflammatory PPARγ agonistic properties (4, 5). Ibuprofen has also been reported to protect against neurodegeneration mediated by reactive oxygen species and glutamate excitotoxicity. So the neuroprotection observed in this study by MMEI can also be attributed to these factors (39, 40). Thus, this study establishes MMEI as a suitable alternative to oral ibuprofen for potential application against PD.

## Conclusion

Findings of the present study demonstrated that optimal Labrafil M 1944CS based mucoadhesive microemulsion using Box-Behnken statistical design becomes an alternative approach for intranasal delivery of ibuprofen. *In-vivo* studies indicated that ibuprofen increased DA concentration significantly in the brain when administered intranasally as mucoadhesive microemulsion. Further, muscular coordination and spontaneous activity of mice significantly improved through nasally applied microemulsion system. Results of immunohistochemistry as direct evidence showed significant increment of TH neuronal count and density of striatal dopaminergic nerve terminals. Thus, it can be concluded that ibuprofen through intranasal mucoadhesive microemulsion may be an effective alternative approach to treat Parkinsonism. 

## References

[1] Aid S, Bosetti F (2011). Targeting cyclooxygenases -1 and -2 inneuroinflammation: Therapeutic implications. Biochimie.

[2] Ali S, Etminan M, Wiens MO, Jafari S (2009). NSAID use and the risk of Parkinson’s disease: Systematic review and meta-analysis of observational studies. Drugs Aging.

[3] Hirsch EC, Hunot S (2009). Neuroinflammation in Parkinson’s disease: A target for neuroprotection. Lancet Neurol.

[4] Smith PF (2008). Inflammation in Parkinson’s disease: An update. Curr. Opin. Investig. Drugs.

[5] Gagne JJ, Power MC (2010). Anti-inflammatory drugs and risk of Parkinson disease: A meta-analysis. Neurology.

[6] Seidl SE, Potashkin JA (2011). The promise of neuroprotective agents in Parkinson’s disease. Front. Neurol.

[7] Summerfield SG, Stevens AJ, Cutler L, Carmen OM, Hammond B, Tang SP, Hersey A, Spalding DJ, Jeffrey P (2006). Improving the in-vitro prediction of in-vivo central nervous system penetration: Integrating permeability, p-glycoprotein efflux, and free fractions in blood and brain. J. Pharmacol. Exp. Ther.

[8] Chen Q, Gong T, Liu J, Wang X, Fu H, Zhang Z (2009). Synthesis, in-vitro and in-vivo characterization of glycosyl derivatives of ibuprofen as novel prodrugs for brain drug delivery. J. Drug Target.

[9] Zhao Y, Qu B, Wu X, Li X, Liu Q, Jin X, Guo L, Hai L, WuY (2014). Design , synthesis and biological evaluation of brain targeting -ascorbic acid prodrugs of ibuprofen with “lock-in” function. Eur. J. Med. Chem.

[10] Bitter C, Suter-Zimmermann K, Surber C (2011). Nasal drug delivery in human. Curr. Probl. Dermatol.

[11] Ugwoke MI, Agu RU, Verbeke N, Kinget R (2005). Nasal mucoadhesive drug delivery: Background, applications, trends and future perspectives. Adv. Drug Deliv. Rev.

[12] Patel MB, Mandal S, Rajesh KS (2013). Formulation and kinetic modeling of Curcumin loaded intranasal mucoadhesive Microemulsion. J. Pharm. Bioall. Sci.

[13] Ghosh PK, Murthy RSR (2006). Microemulsions: A potential drug delivery system. Curr. Drug Deliv.

[14] Fanun M (2010). Microemulsions with nonionic surfactants and mint oil. Open Colloid Sci. J.

[15] Acharya SP, Pundarikakshudu K, Panchal A, Lalwani A (2013). Preparation and evaluation of transnasal microemulsion of carbamazepine. Asian J. Pharm. Sci.

[16] Pathak R, Dash RP, Misra M, Nivsarkar M (2014). Role of mucoadhesive polymers in enhancing delivery of nimodipine microemulsion to brain via intranasal route. Acta Pharm. Sin. B.

[17] Cho HJ, Ku WS, Termsarasab U, Yoon I, Chung CW, Moon HT, Kim DD (2012). Development of udenafil-loaded microemulsions for intranasal delivery: In-vitro and in-vivo evaluations. Int. J. Pharm.

[18] Muhammad N, Nisar UR, Khan JA, Sehti A, Nawaz Z (2013). Development and optimization of microemulsion formulation using Box-Behnken design for enhanced transdermal delivery of Lornoxicam. Lat. Am. J. Pharm.

[19] Mandpe L, Pokharkar V (2013). Quality by design approach to understand the process of optimization of iloperidone nanostructured lipid carriers for oral bioavailability enhancement. Pharm. Dev. Technol.

[20] Hapse SA, Kadaskar PT, Shirsath AS (2011). Difference spectrophotometric estimation and validation of ibuprofen from bulk and tablet dosage form. Pharm. Lett.

[21] Moghimipour E, Salimi A, Leis F (2012). Preparation and evaluation of Tretinoin microemulsion based on Pseudo-ternary phase diagram. Adv. Pharm. Bull.

[22] Chopra S, Motwani SK, Iqbal Z, Talegaonkar S, Ahmad FJ, Khar RK (2007). Optimisation of polyherbal gels for vaginal drug delivery by Box-Behnken statistical design. Eur. J. Pharm. Biopharm.

[23] Mandal S, Mandal SD (2010). Design and development of Carbamazepine mucoadhesive microemulsion for intranasal delivery: An ex-vivo study. Int. J. Pharm. Sci. Rev. Res.

[24] Harris AS, Svensson E, Wagner ZG, Lethagen S, Nilsson IM (1988). Effect of viscosity on particle size, deposition, and clearance of nasal delivery systems containing desmopressin. J. Pharm. Sci.

[25] Lu HT, Chen RN, Sheu MT, Chang CC, Chou PY, Ho HO (2011). Rapid onset Sildenafil nasal spray carried by microemulsion systems: In-vitro evaluation and in-vivo pharmacokinetic studies in rabbits. Xenobiotica.

[26] Muhammad N, Nisar UR, Khan JA, Sehti A, Nawaz Z (2013). Development and optimization of microemulsion formulation using Box-Behnken design for enhanced transdermal delivery of Lornoxicam. Lat. Am. J. Pharm.

[27] Huang CT, Tsai MJ, Lin YH, Fu YS, Huang YB, Tsai YH, Wu PC (2013). Effect of microemulsions on transdermal delivery of Citalopram: Optimization studies using mixture design and response surface methodology. Int. J. Nanomed.

[28] Jagtap P, Jadhav K, Dand N (2015). Formulation and ex-vivo evaluation of solid lipid nanoparticles (SLNs) based hydrogel for intranasal drug delivery. Int. J. Med. Health Biomed. Bioeng. Pharm. Eng.

[29] Sharma G, Mishra AK, Mishra P, Misra A (2009). Intranasal Cabergoline: Pharmacokinetic and Pharmacodynamic studies. AAPS PharmSciTech.

[30] Shiotsuki H, Yoshimi K, Shimo Y, Funayama M, Takamatsu Y, Ikeda K, Takahashi R, Kitazawa S, Hattori N (2010). A rotarod test for evaluation of motor skill learning. J. Neurosci. Methods.

[31] Basso DM, Beattie MS, Bresnahan JC (1995). A sensitive and reliable locomotor rating scale for open field testing in rats. J. Neurotrauma.

[32] Liu GP, Ma Q, Shi N (2006). Tyrosine hydroxylase as a target for deltamethrin in the nigrostriatal dopaminergic pathway. Biomed. Environ. Sci.

[33] Barbiero JK, Santiago RM, Lima MM, Ariza D, Morais LH, Andreatini R, Vital MA (2011). Acute but not chronic administration of pioglitazone promoted behavioral and neurochemical protective effects in the MPTP model of Parkinson’s disease. Behav. Brain Res.

[34] Jayaraj RL, Elangovan N, Manigandan K, Singh S, Shukla S (2014). CNB-001 a novel curcumin derivative, guards dopamine neurons in MPTP model of Parkinson’s disease. BioMed Res. Int.

[35] Oo TF, Kholodilov N, Burke RE (2003). Regulation of natural cell death in dopaminergic neurons of the substantianigra by striatal glial cell line-derived Neurotrophic factor in-vivo. J. Neurosci.

[36] Elshafeey AH, Bendas ER, Mohamed OH (2009). Intranasal microemulsion of sildenafil citrate: In-vitro evaluation and in-vivo pharmacokinetic study in rabbits. AAPS PharmSciTech.

[37] Mandal S, Mandal SD, Surti N, Patel VB (2010). Development of microemulsion formulation for the solubility enhancement of flunarizine. Pharm. Lett.

[38] Makhmalzadeh BS, Torabi S, Azarpanah A (2012). Optimization of ibuprofen delivery through rat skin from traditional and novel nanoemulsion formulations. Iran. J. Pharm. Res.

[39] Yusuke I, Olivier N, David Z, Toru O, Richard AJ (2010). Ibuprofen for neuroprotection after cerebral ischemia. J. Thorac. Cardiovasc. Surg.

[40] Asanuma M, Nishibayashi AS, Miyazaki I, Kohno M, Ogawa N (2001). Neuroprotective effects of non-steroidal anti-inflammatory drugs by direct scavenging of nitric oxide radicals. J. Neurochem.

